# Mixed and blended emotional reactions to 2014 Ebola outbreak

**DOI:** 10.7189/jogh.10.010304

**Published:** 2020-06

**Authors:** Jie Zhuang, Tai-quan (Winson) Peng, Jiliang Tang, Yingcai Wu

**Affiliations:** 1 Department of Communication Studies, Texas Christian University, Fort Worth, Texas, USA; 2 Department of Communication, Michigan State University, East Lansing, Michigan, USA; 3 Computer Science and Engineering Department, Michigan State University, Michigan, USA; 4 College of Computer Science and Technology, Zhejiang University, Hangzhou, Zhejiang Province, P R China

**Figure:**
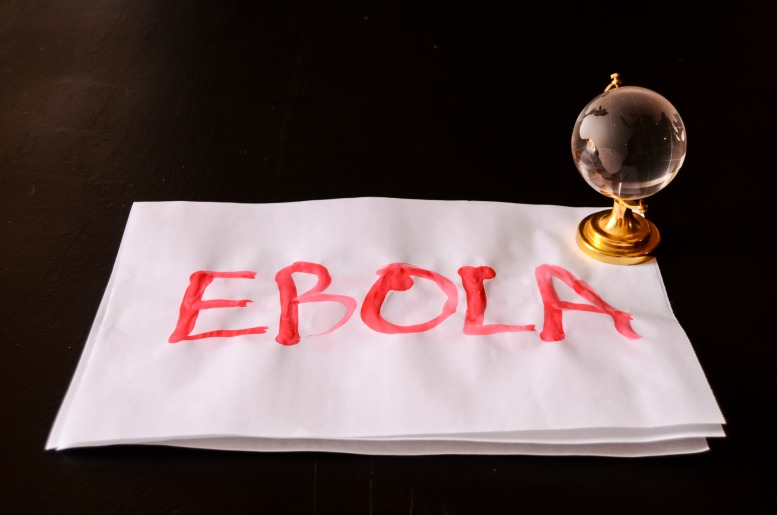
Photo: Source – Shutterstock (https://www.shutterstock.com/image-photo/word-ebola-text-writed-blood-on-1500631940), copyright free.

How individuals emotionally respond to an epidemic has significant impact on their subsequent behavioral responses, ranging from complete ignorance to active engagement in suggested protection behaviors [1]. When facing with health threats, people experience strong emotional feelings and a series of emotional swifts [2], from initial fear of the deadliness of disease and anxiety about one’s possible exposure to the disease, to anger at health systems and governments for their incapability of providing necessary protection, and at particular individuals for transmitting the disease. Sorrow and sadness are also commonly experienced for loss of lives.

Researchers have increasingly begin to examine how people emotionally respond to health threats. For example, by analyzing a large number of online texts in response to Middle East Respiratory Syndrome, researchers found a wide spread of negative emotional expressions (ie, anxiety and fear) [3]. Researchers found a low collective level of anxiety [4] and a higher level of worry [5] toward 2009 Influenza A pandemic in Hong Kong. Emotions are transient and dynamic, that is, people tend to feel a shift from one emotion to another over the course of an emotionally charging stimulus. In the case of 2014 Ebola, people were likely to experience fear due to the deadly consequences of Ebola at its onset in Africa. When mortality rates plateaued and started to decline, quarantined Ebola suspect cases were declared Ebola free and discharged from hospitals, and survival cases appeared, positive emotional responses began to emerge and intertwine with negative emotional responses. Individuals’ arousal of positive and negative emotions may also be influenced by the spatial distance resulted from moving stimuli influenced [6]. In the context of health threats, it is logical to aruge that when people are physically close to a health threat, their feeling of fear is more frequent and stronger in comparison to people who are physically distant.

## A CASE STUDY: EMOTIONAL RESPONSES TO EBOLA OUTBREAK IN 2014

Ebola outbreak in 2014 was unprecedented and caused an astonishing number of death in multiple countries [7].This outbreak provideed an excellent context for studying emotion co-evolution, co-existence, and emotional shift over time and across geographic locations.

The study drew a random sample consisting of 21 000 original tweets from the original tweets across 30 countries (a stratified sampling approach is employed in the study to draw a random sample of original tweets from all the tweets of each country). The retweets of the 21 000 original tweets were extracted, resulting in a study data set of 435 700 tweets including the 21 000 original tweets and their 424 700 retweets. The emotional types embedded in the 21 000 orginal tweets were manually coded. The remaining 424 700 tweets were duplicates or retweets of the 21 000 original ones and did not provide new information. Emotional expressions in the corpus of 424 700 tweets were assessed based on the coding results of their corresponding original tweets. The countries from which tweets were extracted were grouped to a) countries with confirmed cases, b) countries that are geographically adjacent with countries listed in a), and c) countries that are geographically remote.

Expression of emotions embedded in user-generated tweets were manually coded and categorized into positive, negative, and neutral expressions.Negative expressions were decomposed into three discrete negative emotions, fear, anger, and sadness. Discrete emotions with a positive valence were not further differentiated. Fear was coded for when a tweet explicitly mentioned synonyms of fear (afraid, scared, terrified, horrified) or when self-oriented protection was mentioned. Anger was coded for when one or more synonyms of anger (irritated, outraged, mad, upset) was used or a tweet clearly blamed an entity, and sadness was coded for when its synonyms (sorrow, grief) were used or when other-oriented protection was mentioned. Tweets were coded as positively valenced when they explicitly stated happiness, content, satisfaction, encouragement, and their synonyms, and coded as neutral when they only tweeted or re-tweeted news-like headlines without adding further information.

In Twitter, public concern on Ebola was not evenly dstributed across countries [8]. Out of the 435 700 tweets on Ebola, over half of the tweets (52.6%) were posted by users from the United States (52.6%), followed by the United Kingdom (12.7%), Nigeria (4.2%), Canada (3.6%), Indonesia (2.2%), and Australia (2.0%). The data spreaded wide across all 12 months in 2014. There was a substantial rise starting July of 2014, and reached its peak in October 2014. Between March and April 2014, there was a secondary rise in the total amount of tweets.

**Figure 1:**
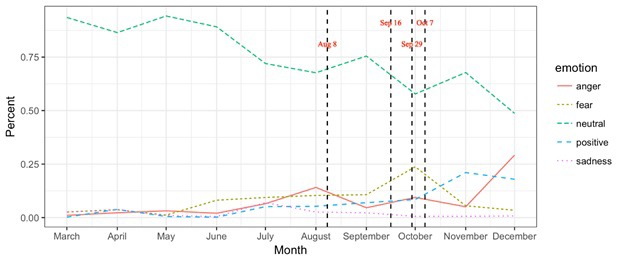
Verbal expressions of emotions on Twitter over the course of 2014 Ebola outbreak.

Out of 435 700 tweets, 62.5% were neutral, 9.6% were positive, and 27.9% contained negative emotions. Among the negatively valenced tweets, 61.3% contained verbal expressions associated with fear, 34.2% contained verbal expressions associated with anger, and 4.5% contained verbal expressions associated with sadness (**Figure 1**).

Tweets with verbal expressions of positive emotion are found to increase at a relatively slow rate. Tweets with verbal expressions of sadness reached its peak in July 2014, which is followed by a monotonic decrease in the subsequent five months. Tweets with verbal expressions of fear reached its singular peak in October 2014, followed by a substantial decrease in November and December. Tweets expressing anger reached its first peak in August 2014 followed by a dramatic decrease in the next three months and then reached its second peak in December 2014 (**Figure 1**).

Twitter users in countries with confirmed Ebola cases generated significantly less amount of verbal expressions of positive emotions (9.1%) than in countries that are geographically adjacent (11.1%) or remote (10.5%) from confirmed Ebola cases. The percent of tweets with verbal expressions of fear generated by countries with confirmed cases (18.1%) was higher than adjacent countries (17.6%) or geographically remote countries (12.4%). The percent of tweets with anger-related expressions generated from countries with confirmed cases (10.5%) was significantly larger than the percent of tweets generated by adjacent countries (8.5%) and distant countries (6%). The percent of tweets expressing sadness generated by countries with confirmed cases (1.2%) was larger than the percent of tweets expressing sadness by adjacent countries (1.1%), but smaller than that by distant countries (1.5%).

Generating a systematic understanding about what emotions are experienced in response to public health threats is necessary as it likely provides signals and evidence for early discovery and timely alarming of emerging health risks [9,10]. The scaricty in research to comprehensively examine how public emotionally respond to health threats warranted this endeavor. It was clear that twitter users used tense emotional expressions as responses to Ebola, and the valence and type of emotions expressed on Twitter were affected by the temporal factors and spatial distance from confirmed Ebola cases.

## FUTURE DIRECTIONS

Despite of generating inspiring findings, this research also calls for further attention to the following research areas. With a vast majority of the data coming from the US, findings generated from this research limit our ability to make more geralized claims regarding how Twitter users from other countries verbally express emotional reactions to Ebola outbreak. Future research is needed to examine whether Twitter users from other countries verbally expressions emotions toward Ebola in a similar or dissimilar manner.

As a descriptive study, this research did not examine the impact of collective mixed and blended emotions on cognitive and behavioral outcomes. Future research is desired to examine these relationships. This research was limited to the scope of only analyzing tweets without reference to external information. Information like news release from external sources and talks delivered by government officials might serve as additional stimuli to the Twitter system and hence exert impact on how Twitter users emotionally respond to Ebola. Future research is needed to close this gap.

The present research has several practical implications. First, this research provided a landscape for emotional responses to 2014 global Ebola outbreak by simultaneously examining discrete negative and positive emotions at a collective level. Second, precisely detecting how the public emotionally feel about a health threat not only offers policy makers and health practioners opportunities to adequately address public concerns and inform the public about health risks.

## CONCLUSION

To conclude, one cannot stress enough about the powerful impact of emotions on employed actions to cope with health threats. A better understanding about how the public respond to health threats such as Ebola emotionally will enable health campaign practitioners to design campaigns and education materials that address fear, assuage anger, and deliver optimism, hope, and empowerment.
